# Expression of NGF and GDNF family members and their receptors during peripheral nerve development and differentiation of Schwann cells *in vitro*

**DOI:** 10.1016/j.neulet.2009.11.060

**Published:** 2010-01-18

**Authors:** Marko Piirsoo, Anne Kaljas, Karin Tamm, Tõnis Timmusk

**Affiliations:** Department of Gene Technology, Tallinn University of Technology, Akadeemia tee 15, 12618 Tallinn, Estonia

**Keywords:** Neurotrophins, GDNF family of neurotrophic factors, Sciatic nerve, Schwann cell

## Abstract

Ligands of NGF and GDNF families of neurotrophic factors have important functions in the development of the vertebrate peripheral nervous system (PNS). It has been established that they also play key roles in the regeneration of PNS. Expression patterns of NGF and GDNF family members and their receptors have mostly been analyzed during regeneration, and less during development of the PNS. We describe the expression of mRNAs encoding these neurotrophic factors and their receptors during development of rat sciatic nerve and in three modes of differentiation of cultured rat Schwann cells. Our results demonstrate specific expression patterns of NGF and GDNF family ligands and their receptors during differentiation of Schwann cells *in vivo* and *in vitro*.

## 1 Introduction

Members of both NGF and GDNF families of neurotrophic factors are important in the development of the vertebrate PNS [3,2]. Neurotrophins BDNF and NGF are important positive regulators of myelination by Schwann cells, whereas NT-3 negatively regulates myelination in PNS. Exogenous administration of BDNF enhances myelination in PNS, whereas removal of endogenous BDNF inhibits myelination in mice [6,26]. Positive effect of BDNF on myelination in PNS comes through binding of BDNF to p75NTR receptor [8]. In addition, BDNF is required for re-myelination in the injured peripheral nerve in rodents *in vivo* [29]. It has been suggested that the major source of BDNF supporting myelination in DRG and Schwann cell co-cultures is neuronal cells [22]. NGF promotes myelination in DRG and Schwann cell co-cultures, whereas blocking NGF activity *in vivo* inhibits myelination [7]. GDNF promotes myelination of small caliber axons that normally do not myelinate and enhances myelination in neuron-Schwann cell co-cultures [12,14]. In contrast, NT-3 negatively regulates myelination *in vivo* and this effect is achieved through binding of NT-3 to its high affinity receptor NTRK3 [6].

Schwann cells are the major macroglial cells in PNS. They ensheath axons and, depending on axon caliber, differentiate to either myelinating or non-myelinating Schwann cells. Upon peripheral nerve injury they de-differentiate, start to proliferate, support regeneration of the peripheral nerve and re-differentiate upon axonal regeneration (reviewed in Ref. [15]). Expression of NGF and GDNF family members is regulated in the injured nerve with specific time course for each gene [18,11,27]. In cell culture rat Schwann cells proliferate in the presence of neuregulin and low concentrations of forskolin. Upon elevated forskolin levels, Schwann cells differentiate and induce the expression of myelin genes [21]. GDNF and BDNF are the predominant neuregulin-releasing neurotrophic factors produced by cultured Schwann cells [9].

Despite the fact that neurotrophic factors have important roles in PNS development, expression patterns of the factors during nerve development has not been studied thoroughly. In the present study we have analyzed the expression of NGF and GDNF family members and their receptors during rat sciatic nerve development and in three differentiation stages of cultured rat Schwann cells.

## 2 Materials and methods

Rat Schwann cell (SC) cultures from postnatal day 3 rat pups were established as described [5]. Pure SC culture was maintained and passaged in DMEM, 3% FCS, 2 μM forskolin and 5% neuregulin-beta conditioned medium in Primaria (Clontech) tissue culture dishes. In the differentiation experiments the medium was replaced with defined medium (DMEM/F12, 1×N2 supplement and 5% neuregulin-beta conditioned medium) and incubated overnight. Next day the medium was replaced with defined medium containing 20 μM forskolin (Sigma) to induce the differentiation of Schwann cells. The growth-arrested Schwann cells were grown in defined medium containing 0.2% DMSO to match the DMSO concentration in the culture media of differentiated cells, treated with forskolin dissolved in DMSO. Thirty-six hours later the cells were used for subsequent analysis. Cell cycle profiles were measured by propidium iodide staining and BrdU incorporation using cell sorter FacsCalibur (Becton Dickinson) [24].

Total RNA from rat tissues and Schwann cells was extracted using RNAWiz (Ambion). First-strand cDNAs were synthesized with Superscript III (Invitrogen) reverse transcriptase. PCR reactions were performed using 1/50 of the first-strand cDNA reaction and the PCR products were analyzed in the exponential phase of amplification. Primers used are depicted in Supplementary Table 1. Real-time quantitative PCR (qPCR) analysis was performed in triplicates using qPCR Core Kit for SYBR Green I (Eurogentec) with Lightcycler 2.0 (Roche) (Supplementary Table 2). Schwann cell data was normalized with housekeeping gene HPRT. Data was analyzed and standard deviation (SD) calculated as described [4]. Exon-specific BDNF primers have been previously described [1]. BDNF protein levels were measured in cell homogenates using ChemiKine Brain Derived Neurotrophic Factor Sandwich Elisa Kit (Chemicon).

## 3 Results and discussion

### 3.1 Expression of neurotrophins and their receptors during rat peripheral nerve development and in cultured Schwann cells

**Fig. 1:**
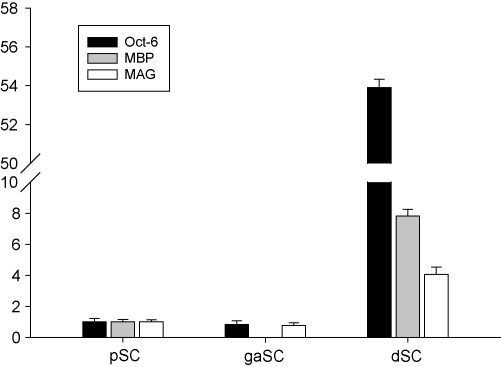
Quantitative RT-PCR analysis of expression of Schwann cell markers Oct-6, MBP and MAG mRNAs in proliferating (pSC), growth-arrested (gaSC) and differentiated (dSC) Schwann cells. Expression levels are represented relative to the level of the respective mRNA in pSC, which was arbitrarily set at one.

**Fig. 2:**
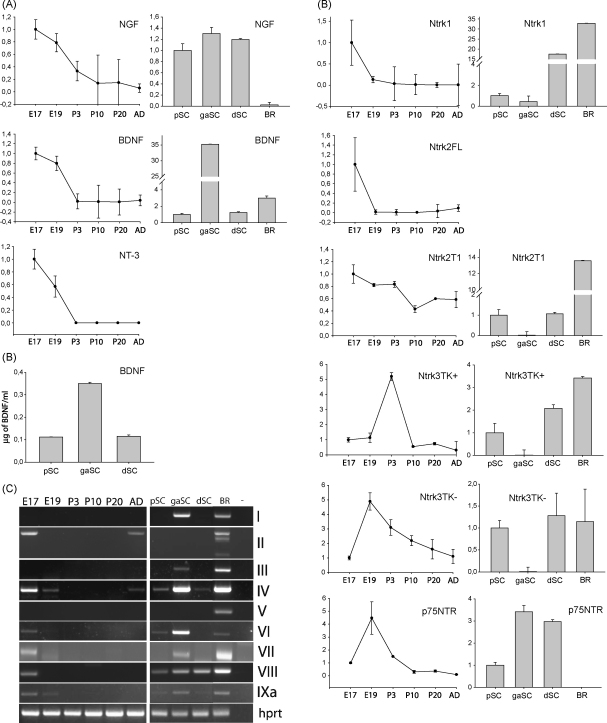
(A) Quantitative RT-PCR analysis of expression of neurotrophin mRNAs during rat sciatic nerve development and in three modes of Schwann cell differentiation. (B) Analysis of BDNF protein levels in three modes of Schwann cell differentiation using ELISA. (C) Semiquantitative RT-PCR analysis of expression of BDNF 5′ exon-specific mRNAs during rat sciatic nerve development and in three modes of Schwann cell differentiation. (D) Quantitative RT-PCR analysis of expression of neurotrophin receptor mRNAs during rat sciatic nerve development and in three modes of Schwann cell differentiation. Expression levels are represented relative to the level of the respective mRNA at E17 (in sciatic nerve development) or in pSC (in Schwann cells), which was arbitrarily set at one. pSC: proliferating Schwann cells, gaSC: growth-arrested Schwann cells, dSC: differentiated Schwann cells, BR: adult rat brain.

In cultured Schwann cells neurotrophin expression was analyzed in cells growing under three different conditions. Proliferating and growth-arrested Schwann cells expressed low levels of differentiation marker Oct-6 and myelin genes MBP and MAG. Expression of Schwann cell differentiation marker Oct-6 and myelin genes MBP and MAG mRNAs was induced in differentiated cells (Fig. 1). Proliferating Schwann cells exhibited normal cell cycle profile with 53.4% of the cells in G1, 30.3% in S and 15.5% in G2/M phase of the cell cycle as measured by propidium iodide staining and BrdU incorporation. Growth-arrested and differentiated Schwann cells did not proliferate with 87.4 and 88.2% of the cells arrested in G1 phase of the cell cycle respectively (Supplementary Fig. 1). NGF mRNA was expressed during all three modes of Schwann cell differentiation at 50-fold higher levels than in adult rat brain. BDNF mRNA was expressed at 35- and 20-fold higher level in growth-arrested Schwann cells than in proliferating and differentiated cells respectively (Fig. 2A). The 35-fold induction of BDNF mRNA levels were accompanied by a 3-fold increase in BDNF protein levels in growth-arrested cells as compared to proliferating and differentiated cells (Fig. 2B). We did not detect NT-3 and NT-4 expression in cultured Schwann cells (data not shown).

We analyzed NGF, BDNF, NT-3 and NT-4 mRNA expression during rat sciatic nerve development at embryonic day (E) 17, E19, postnatal day (P) 3, P10, P20 and P60 by qPCR analysis. In general, NGF, BDNF and NT-3 expression levels were higher in embryonic sciatic nerves and downregulated upon nerve development. Expression levels of NGF, BDNF and NT-3 mRNAs were highest at E17, the earliest developmental stage analyzed, decreased slightly at E19 and were markedly downregulated during postnatal nerve development (Fig. 2A). We could not detect NT-4 mRNA expression in rat sciatic nerves (data not shown).

Expression of neurotrophins in the postnatal rodent sciatic nerve has been shown previously [18,11]. Our results demonstrating that neurotrophin expression is high in embryonic sciatic nerves and the levels are decreasing during development suggests two possible scenarios. First, it is possible that Schwann cells in the embryonic nerves are not a homogenous population of glial cells, but rather the phenotype of a Schwann cell depends on the type of axon it is contacting with. This means that during late embryogenesis different Schwann cell populations express different set of neurotrophins. This has also been suggested by a study showing that Schwann cells in the regenerating peripheral nerve exhibit distinct sensory and motor phenotypes [13]. Alternatively it is possible that embryonic Schwann cells exhibit homogenous phenotype, but express higher level of neurotrophins to provide maximal support for growing axons.

Structure of BDNF gene is complex as compared to other neurotrophins. Rat BDNF gene contains nine 5′ noncoding exons and one coding exon. Each noncoding exon has its own promoter and is differentially expressed in the nervous system and in other tissues. The 5′ noncoding exons fall into two clusters, with exons I–III as a 5′ cluster and exons IV–VIII 3′ cluster. In addition, a separate promoter is driving expression of exon IXa transcripts initiating in the 5′ region of exon IX [1]. Semiquantitative RT-PCR analysis showed that BDNF exons II, IV, VI–IXa mRNAs were expressed in sciatic nerve, with highest levels at E17, the first developmental stage analyzed (Fig. 2C) similarly to the levels of total BDNF mRNA (Fig. 2A). In the adult sciatic nerve the expression of exons II and IV mRNAs was induced indicating that the slight upregulation of BDNF mRNA expression there could be accounted to the expression from BDNF promoters II and IV (Fig. 2C).

In cultured Schwann cells BDNF exons I, III, IV, VI–IXa mRNAs were expressed and the levels were highest in growth-arrested Schwann cells similarly to total BDNF mRNA (Fig. 2C). Exon VIII mRNA was expressed at lower level in proliferating Schwann cells and at higher level in growth-arrested and differentiated Schwann cell suggesting that the slight increase in the expression of BDNF in differentiated as compared to proliferating Schwann cells observed in real-time PCR analysis could be attributed to exon VIII expression. The fact that we detected BDNF exon II expression in developing sciatic nerve but not in cultured Schwann cells suggests that BDNF exon II is expressed in fibroblasts or microglial cells of the developing sciatic nerve. Alternatively, exon II transcripts are expressed in Schwann cells *in vivo* but not *in vitro*. Our analysis also showed that BDNF exon III was expressed only in growth-arrested Schwann cells but not in the sciatic nerve suggesting that cell culture conditions we used promoted ectopic expression of BDNF exon III in Schwann cells.

Next we investigated expression of receptors of neurotrophins during sciatic nerve development. Expression of Ntrk1 (TrkA) mRNA, coding the high affinity receptor for NGF, was highest at E17, decreased more than 5-fold at E19 and was almost undetectable postnatally (Fig. 2D). Previous reports have found that Ntrk1 is not expressed in the developing or regenerating rat sciatic nerve [11,23]. It is possible that sensitivity of analyses did not allow the detection of Ntrk1 expression in these studies. Ntrk2 (TrkB), the high affinity receptor for BDNF and NT-4 has three major isoforms [16,19]. Full-length kinase domain containing Ntrk2FL is mainly expressed in neuronal cells, whereas kinase deficient T1 and T2 isoforms are expressed in glial cells [10]. Ntrk2FL mRNA levels were highest in E17 sciatic nerve, 70-fold decreased at E19, undetectable during early postnatal stages and upregulated during later development (Fig. 2D). Ntrk2T1 mRNA level was highest at E17, and decreased thereafter reaching 2-fold lower levels in adult sciatic nerve (Fig. 2D). We did not detect Ntrk2T2 expression in the sciatic nerve (data not shown). Our results are consistent with previously published data about postnatal expression of NtrkT1 and lack of Ntrk2T2 protein expression in the sciatic nerve [8]. It has also been shown that Ntrk2FL is expressed at low level in adult rat sciatic nerve [8]. Here we show that, in addition to this, full-length Ntrk2 mRNA is expressed in E17 embryonic nerves. Ntrk3 (TrkC), the high affinity receptor for NT-3, has two major isoforms, full-length Ntrk3 harboring tyrosine kinase domain (TK+) and truncated Ntrk3 (TK−) [19]. It has been previously shown that Ntrk3 mRNA is expressed in embryonic rat sciatic nerve from E14 to E18 [17]. Our results showed that Ntrk3TK+ mRNA was expressed at E17 and E19 at similar levels, the levels increased 5-fold at P3, decreased 10-fold at P10 and remained unchanged during later postnatal development (Fig. 2D). Ntrk3TK− mRNA level was upregulated 5-fold at E19 as compared to E17 and gradually decreased during postnatal development (Fig. 2D). This contrasts to previously published data showing that Ntrk3 protein level is gradually downregulated during postnatal development of rat sciatic nerve [8], suggesting that Ntrk3 mRNA and protein levels are regulated in a different manner. Expression of p75NTR mRNA, the low-affinity neurotrophin receptor, increased 2.5-fold from E17 to E19 and was downregulated later during development (Fig. 2D). This is consistent with published data showing that p75 expression is downregulated during postnatal development [8]. We show here for the first time that its expression is even higher in embryonic sciatic nerves.

Analysis of expression of neurotrophin receptors in cultured Schwann cells showed that in differentiated cells Ntrk1 mRNA level was 17 times higher than in proliferating cells and 34 times higher than in growth-arrested cells and 2 times lower than in adult rat brain (Fig. 2D). Full-length Ntrk2 and Ntrk2T2 were not expressed in cultured Schwann cells (data not shown). Ntrk2T1, Ntrk3TK+ and Ntrk3TK− mRNA was detected in proliferating and in differentiated Schwann cells, but not in growth-arrested cells. Ntrk2T1 mRNA was expressed in proliferating and differentiated Schwann cells at similar level that was 14 times lower than in adult rat brain (Fig. 2D). Expression of Ntrk3Tk+ mRNA was induced 2-fold in differentiated cells as compared to proliferating cells and the level was 3.5 times lower than in rat brain. Expression level of Ntrk3TK− was similar in proliferating and differentiated Schwann cells and in adult rat brain. Expression of p75NTR mRNA was induced 3.5- and 3-fold in growth-arrested and differentiated Schwann cells respectively as compared to proliferating cells where the levels were still more than 300 times higher than in adult rat brain (Fig. 2D).

### 3.2 Expression of GDNF family of neurotrophic factors and their receptors during rat peripheral nerve development and in cultured Schwann cells

**Fig. 3:**
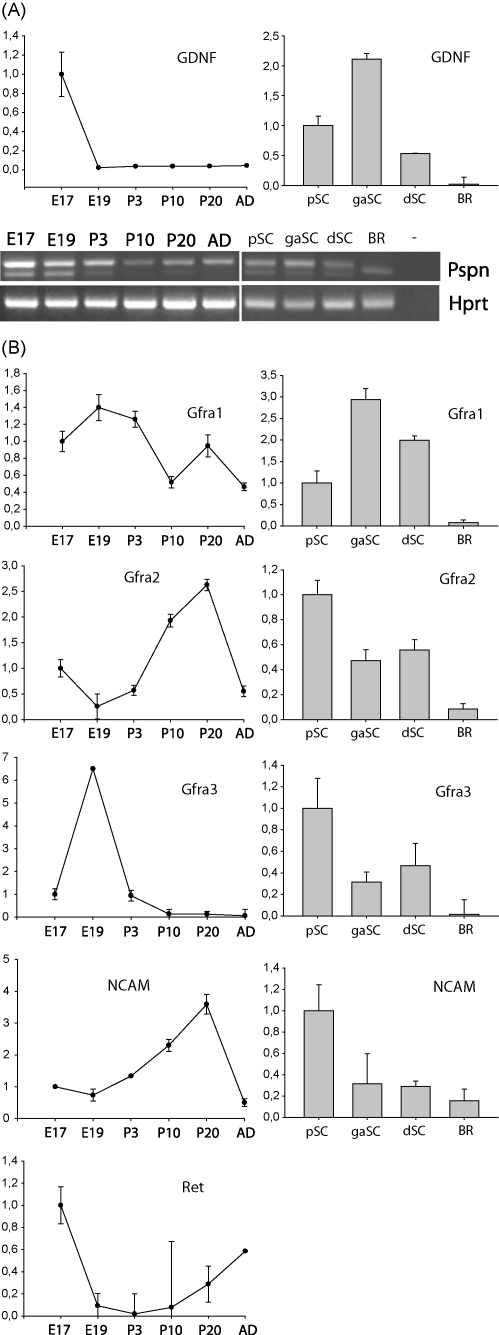
(A) Quantitative and semiquantitative RT-PCR analysis of GDNF and Pspn mRNA expression during rat sciatic nerve development and in three modes of Schwann cell differentiation. (B) Quantitative RT-PCR analysis of expression of receptors for GDNF family members during rat sciatic nerve development and in three modes of Schwann cell differentiation. Expression levels are represented relative to the level of the respective mRNA at E17 (in sciatic nerve development) or in pSC (in Schwann cells), which was arbitrarily set at one. pSC: proliferating Schwann cells, gaSC: growth-arrested Schwann cells, dSC: differentiated Schwann cells, BR: adult rat brain.

Next we analyzed GDNF, Artn, Pspn and Nrtn gene expression during rat sciatic nerve development (Fig. 3A). GDNF mRNA level was highest at E17, decreased more than 30-fold at E19 and remained almost undetectable later during development. It has been previously shown that GDNF is expressed in the distal part of the lesioned adult rat sciatic nerve and in embryonic chick sciatic nerves [27,9]. Here we show that expression of GDNF is relatively high in rat embryonic sciatic nerve and is downregulated upon nerve maturation. Pspn is expressed in two forms in mammals. Majority of the Pspn mRNA has intron retention, leading to frame-shift in the open reading frame and the minor form encodes biologically active protein [20]. Expression of the shorter functional Pspn mRNA was high at E17 and E19 and decreased postnatally to undetectable levels. As shown previously, Pspn mRNA with intron retention had higher expression level than the spliced mRNA and was detected at all stages during sciatic nerve development, with highest levels at E17 and E19 (Fig. 3A). We did not detect Nrtn and Artn mRNA expression in sciatic nerve (data not shown).

Analysis of GDNF expression in cultured Schwann cells showed that in growth-arrested cells the levels were 2-fold higher than in proliferating cells, and 4-fold higher than in differentiated cells (Fig. 3A). Spliced Pspn mRNA was expressed at similar level in all three modes of cultured Schwann cells (Fig. 3A). Nrtn and Artn expression was not detected in cultured Schwann cells (data not shown).

Next, qPCR analysis was performed to investigate expression of receptors for GDNF family of neurotrophic factors (Fig. 3B). It has previously been shown that Gfra1 is expressed in adult sciatic nerve and its expression is upregulated in the distal part of lesion after nerve injury [27]. All members of GFR alpha receptor family, except Gfra4, were expressed in rat sciatic nerve and in cultured Schwann cells (Fig. 3B). The levels of Gfra1 mRNA increased slightly from E17 to E19, were not changed at P3 and decreased during later development. In cultured Schwann cells Gfra1 mRNA was induced 3- and 2-fold in growth-arrested and in differentiated cells respectively as compared to proliferating cells, where the levels were more than 12 times higher than in adult rat brain. Gfra2 mRNA level decreased 4-fold at E19 as compared to E17, thereafter the levels increased 10-fold up to P20 and were decreased again in adult sciatic nerve. Gfra3 mRNA level was induced 6-fold at E19 as compared to E17, decreased 6-fold at P3 and remained relatively unchanged thereafter. In cultured Schwann cells Gfra3 mRNA level decreased more than 2-fold in growth-arrested and differentiated cells compared to proliferating cells where it was more than 70 times higher than in adult rat brain. Using in situ hybridization it has previously been shown that Gfra3 mRNA is expressed at similar level in sciatic nerve during embryonic development [28]. In contrast, our results showed Gfra3 mRNA levels increase 6-fold from E17 to E19 (Fig. 3B). In accordance with previously published data we detected no Ret mRNA expression in cultured Schwann cells [27]. However, in the developing sciatic nerve Ret mRNA was expressed, the levels were highest at E17, decreased 10-fold at E19, remained relatively similar up to P3 and increased thereafter (Fig. 3B). It has been shown that GDNF family of neurotrophic factors can also signal through neural cell adhesion molecule NCAM [25]. Therefore, we analyzed NCAM expression in sciatic nerve and in Schwann cells (Fig. 3B). NCAM mRNA was expressed at similar level at E17 and E19, thereafter the levels increased gradually up to P20. In the adult sciatic nerve NCAM mRNA levels were decreased to 2-fold lower levels than at E17. In cultured Schwann cells, NCAM mRNA expression levels were 3-fold higher in proliferating Schwann cells than in growth-arrested and differentiated cells.

Taken together, our results show that all neurotrophins, except NT-4, are expressed during rat sciatic nerve development and the levels decrease starting from E17, the first developmental stage analyzed. Cultured Schwann cells express NGF and BDNF, but not NT-3 and NT-4. BDNF expression is dramatically induced in growth-arrested Schwann cells, while NGF levels do not change significantly in the analyzed three modes of cell culture. All receptors for neurotrophins are expressed in rat sciatic nerve. Cultured Schwann cells express all receptors for neurotrophins except full-length Ntrk2. Two members of GDNF family of neurotrophic factors, GDNF and persephin, are expressed in rat sciatic nerve and in cultured Schwann cells. All Gfra receptors, except Gfra4, are expressed in cultured Schwann cells and in sciatic nerve. Ret and NCAM coreceptors are expressed in sciatic nerve, but only NCAM is expressed in cultured Schwann cells.
