# Supplementary material for: Expression of NGF and GDNF family members and their receptors during peripheral nerve development and differentiation of Schwann cells in vitro
Source: Neurosci Lett. 2010 Jan 18;469(1):135–40. doi: 10.1016/j.neulet.2009.11.060 (PMC2808476; doi:10.1016/j.neulet.2009.11.060)
Supplement: Supplementary file 1 [file mmc1.doc]

**Supplementary table 1.**

Oligonucleotides used in this study.

| Oct6sense | GGTAATGTGTTCTCGCAGAC |
| --- | --- |
| Oct6antisense | CTTGTTGAGCAGCGGTTTGA |
| MBPsense | GCAGAGGACCCAAGATGAAA |
| MBPantisense | GAGGGGGTGTACGAGGTGT |
| MAGsense | ATCATCAACACCCCCAACAT |
| MAGantisense | CACCATGCAGCTGACCTCTA |
| NGFsense | CCCGAATCCTGTAGAGAGTGG |
| NGFantisense | GACAAAGGTGTGAGTCGTGG |
| BDNFsense | GGCCCAACGAAGAAAACCAT |
| BDNFantisense | TCTCACCTGGTGGAACTTTTTCA |
| NT3sense | ACCACGGAGGAAACGCTATG |
| NT3antisense | ATGGCTGAGGACTTGTCGGTC |
| NTRK1sense | GGGTCAGGGACTAGTGGTCA |
| NTRK1antisense | GAACTTGCGGTAGAGGATGC |
| NTRK2FLsense | CATTGGGATGACCAAGATCC |
| NTRK2FLantisense | TTCTCCAAGCTCCCTCTTCA |
| NTRK2T1sense | GTTGGCGAGACATTCCAAGT |
| NTRK2T1antisense | GTCCCAGGAGTTCAGCTCAC |
| NTRK3sense | TAAGCCCCACCAAAGACAAG |
| NTRK3antisense | TCCCTCTGGAAATCCTTCCT |
| NTRK3altsense | GAACCTGAGGTCCAGAGTGG |
| NTRK3altantisense | CCATGGTTAAGAGGCTTGGA |
| P75sense | CATCTCTGTGGACAGCCAGA |
| P75antisense | CTCTACCTCCTCACGCTTGG |
| GDNFsense | GCCGCCGAAGACCACTCCCTC |
| GDNFantisense | GTGCCGCCGCTTGTTTATCTGG |
| PSPNsense | ATGGCTGCAGGAAGACTTCG |
| PSPNantisense | TCATCAAGGAAGGTCACATCA |
| rGFRA1sense | CAATGTGTCGGGTAGCACAC |
| rGFRA1antisense | TTTGTGGTTATGTGGCTGGA |
| GFRa2sense | TCACTGCCAGATGACCTCAG |
| GFRa2antisense | TGTCGTGAGCTCTGTGAAGC |
| rGFRA3sense | TGCGTTTCCACAGACAACTC |
| rGFRA3antisense | AGACAGAACGGGTAGCCTGA |
| rRetsense | CGGACAAGAGGCCAGTATTT |
| rRetantisense | GCAGCCAGGTCCAAGTAGT |
| NCAMsense | AAGGCAGCTCACTTCGTGTT |
| NCAMantisense | ACAATGAGGATGCCCACAAT |
| HPRT1sense | CAGTCCCAGCGTCGTGATTA |
| HPRT1antisense | AGCAAGTCTTTCAGTCCTGTC |

**Supplementary table 2.**

Levels of expression of neurotrophic factors and their receptors measured by quantitative RT-PCR analysis. Graphic representation of the data is shown in figures 2 and 3 of the article. Expression levels are represented relative to the level of the respective mRNA at E17 (in sciatic nerve development) or in pSC (in Schwann cells), which was arbitrarily set at one.

Sciatic nerve

|  | E17 | E19 | P3 | P10 | P20 | AD |
| --- | --- | --- | --- | --- | --- | --- |
| NGF | 1,00 | 0,79 | 0,33 | 0,14 | 0,15 | 0,06 |
| BDNF | 1,00 | 0,80 | 0,02 | 0,01 | 0,01 | 0,04 |
| NT-3 | 1,00 | 0,57 | 0,00 | 0,00 | 0,00 | 0,00 |
| Ntrk1 | 1,00 | 0,13 | 0,04 | 0,01 | 0,01 | 0,01 |
| Ntrk2 FL | 1,00 | 0,01 | 0,01 | 0,01 | 0,03 | 0,09 |
| Ntrk2 T1 | 1,00 | 0,82 | 0,83 | 0,43 | 0,60 | 0,59 |
| Ntrk3 TK+ | 1,00 | 1,14 | 5,19 | 0,55 | 0,75 | 0,32 |
| Ntrk3 TK- | 1,00 | 4,89 | 3,09 | 2,19 | 1,60 | 1,11 |
| p75 NTR | 1,00 | 4,48 | 1,49 | 0,31 | 0,37 | 0,10 |
|  |  |  |  |  |  |  |
| GDNF | 1,00 | 0,02 | 0,04 | 0,04 | 0,04 | 0,05 |
| GfrA1 | 1,00 | 1,40 | 1,26 | 0,52 | 0,95 | 0,46 |
| GfrA2 | 1,00 | 0,26 | 0,57 | 1,93 | 2,63 | 0,55 |
| GfrA3 | 1,00 | 6,50 | 0,94 | 0,14 | 0,13 | 0,06 |
| NCAM | 1,00 | 0,74 | 1,34 | 2,30 | 3,59 | 0,50 |
| Ret | 1,00 | 0,09 | 0,02 | 0,08 | 0,29 | 0,59 |

Cultured Schwann cells

|  | pSC | gaSC | dSC | rat brain |
| --- | --- | --- | --- | --- |
| NGF | 1,00 | 1,30 | 1,19 | 0,02 |
| BDNF | 1,00 | 35,22 | 1,24 | 2,99 |
| Ntrk1 | 1,00 | 0,44 | 17,45 | 32,83 |
| Ntrk2 T1 | 1,00 | 0,02 | 1,07 | 13,58 |
| Ntrk3 TK+ | 1,00 | 0,01 | 2,07 | 3,42 |
| Ntrk3 TK- | 1,00 | 0,01 | 1,28 | 1,14 |
| p75 NTR | 1,00 | 3,42 | 2,98 | 0,00 |
|  |  |  |  |  |
| GDNF | 1,00 | 2,11 | 0,53 | 0,02 |
| GfrA1 | 1,00 | 2,94 | 2,00 | 0,08 |
| GfrA2 | 1,00 | 0,47 | 0,56 | 0,08 |
| GfrA3 | 1,00 | 0,31 | 0,47 | 0,01 |
| NCAM | 1,00 | 0,31 | 0,29 | 0,16 |

**
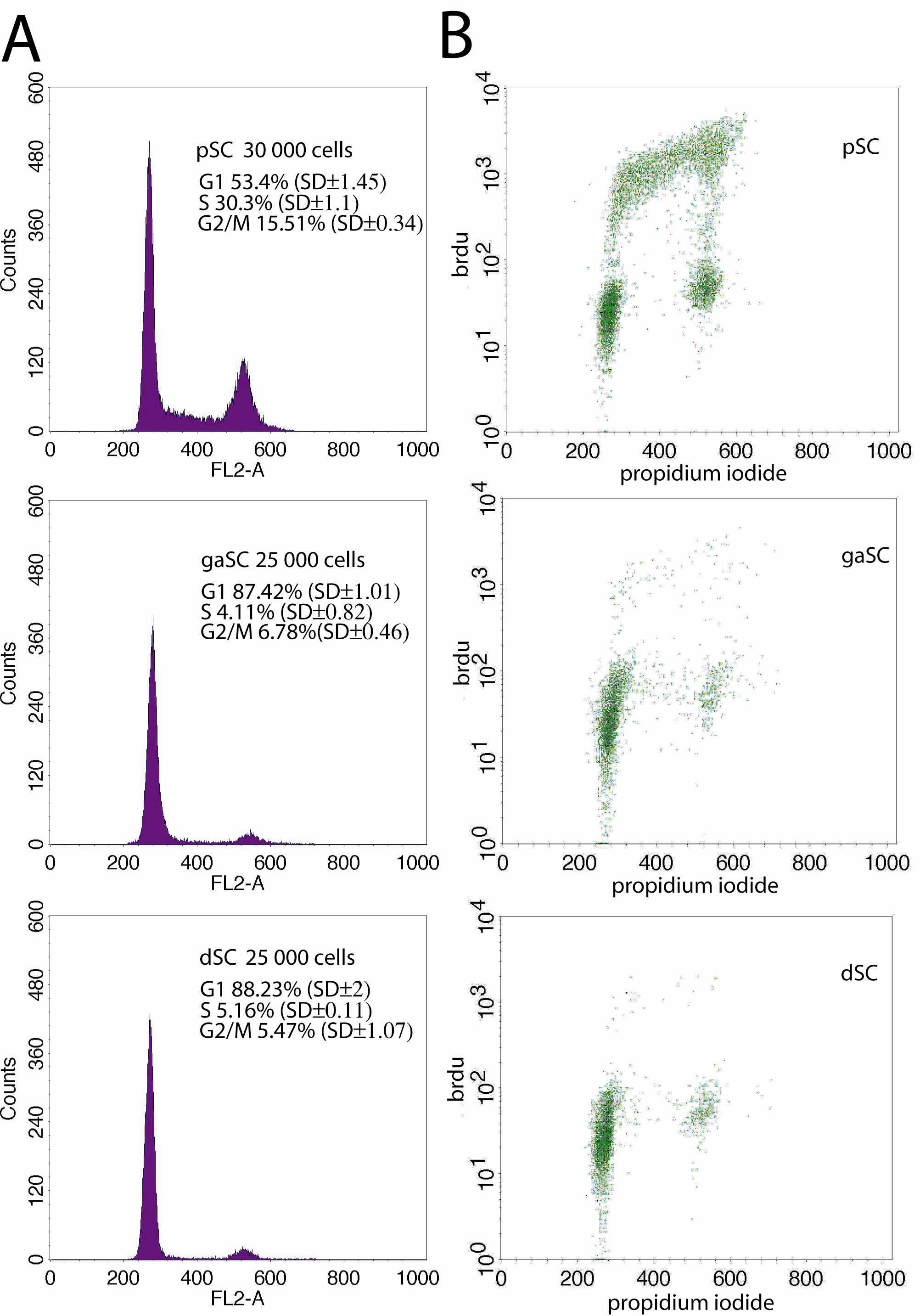
**

**Supplementary figure 1. Cell cycle status of proliferating (pSC), growth arrested (gaSC) and differentiated (dSC) Schwann cells.**

A) Propidium iodide staining of pSC, gaSC and dSC. Histogram plot of DNA content in cells distinguishes between cells in G1, S and G2/M phase of the cells cycle. SD, standard deviation.

B) 5-bromo-2-deoxyuridine (brdu) incorporation in pSC, gaSC and dSC. Dot plot of correlation between intensity of brdu and propidium iodide signals shows the incorporation of brdu into DNA in cells situated in G1, S or G2/M phases of cell cycle. Cells were grown in the presence of brdu for 1 hour, fixed, stained with brdu antibody, followed by staining with FITC labeled secondary antibody and analyzed using FacsCalibur (Becton Dickinson).
